# P-835. Testing on autopilot: Utilization of urine legionella antigen for patients with community-acquired pneumonia

**DOI:** 10.1093/ofid/ofaf695.1043

**Published:** 2026-01-11

**Authors:** Toobah N Wali, Andy H Lim, Elaine H Hassan, Brian H Tong, Lina H Loaiza, Muhammad Fahimuddin, Susanne Burger, Cesar G Berto, Christina Coyle, Michell Huang, Heidi Chen

**Affiliations:** Jacobi Medical Center, Bronx, New York; Jacobi Medical Center, Bronx, New York; Jacobi Medical Center, Bronx, New York; Jacobi Medical Center, Bronx, New York; North Central Bronx, Bronx, New York; Jacobi Medical Center, Bronx, New York; North Central Bronx, Bronx, New York; The University of Alabama at Birmingham, Birmingham, AL; Albert Einstein College of Medicine, Bronx, NY; St. John's University, Bronx, New York; St. John's University, Bronx, New York

## Abstract

**Background:**

Infectious Diseases Society of America (IDSA) and American Thoracic Society (ATS) recommend performing Legionella urinary antigen (LuAg) test only for patients with severe community-acquired pneumonia (CAP) or environmental exposures; however, LuAg is frequently used routinely, regardless of CAP severity. There is an availability of an electronic CAP order set designed to guide appropriate management of CAP. This study aims to assess LuAg ordering practices and compliance to IDSA/ATS CAP guidelines in a large city hospital and the order set.

**Methods:**

A retrospective chart review was conducted on CAP patients who underwent LuAg testing during the first seven days of each month from May 2024 to January 2025, to account for seasonality. CAP severity was classified as per IDSA/ATS guidelines. Inappropriate LuAg testing was defined as testing performed in non-severe pneumonia (NSP) patients.

**Results:**

Of 110 patients, 51% were female and 83% had NSP. Most patients (62%) were admitted to a medicine floor and 26% to an intensive care unit (Table 1). All LuAg resulted negative. Most LuAg tests were ordered by medicine providers (68% in NSP vs 12% in severe CAP; Table 2). The mean duration of Legionella-specific antibiotics was similar in both the NSP and the severe CAP group (1.7 days and 1.8 days, respectively). Inappropriate LuAg ordering occurred for 91 NSP patients, with 82% ordered by medicine providers.

**Conclusion:**

The majority of patients with CAP are admitted to the medicine service. Our findings highlight a lack of adherence to IDSA/ATS CAP guidelines regarding LuAg testing in patients with NSP. No difference in length of Legionella-specific antibiotics was found between those with severe CAP and NSP. The cost of LuAg ranges from $32-$36 per test. This suggests LuAg does not impact clinical management and instead, increases unnecessary diagnostic costs. The overuse of LuAg testing occurred despite the availability of an electronic CAP order set designed to guide appropriate management. Next steps in this project will be education and real-time individual feedback by the antimicrobial stewardship team.

**Disclosures:**

Cesar G. Berto, MD, Basilea: Grant/Research Support|Cidara: Grant/Research Support

